# Methodological challenges and pragmatic design in randomised controlled trials comparing surgery and rehabilitation

**DOI:** 10.1186/1745-6215-16-S2-P172

**Published:** 2015-11-16

**Authors:** Vandana Ayyar Gupta, Antony Palmer, Ines Rombach, Susan Dutton, Cushla Cooper, Karen Barker, David Beard, Sion Glyn-Jones

**Affiliations:** 1 Nuffield Department of Orthopaedics, Rheumatology and Musculoskeletal Sciences, University of Oxford, Oxford, UK; 2 Oxford University Hospitals NHS Trust, Oxford, UK

## 

Randomised controlled trials (RCTs) evaluating surgery versus rehabilitation pose particular methodological and practical challenges. In addition to the differences in the interventions, factors associated with the differences in care pathways and fluctuating symptoms impact the feasibility of effectively running a trial in a hospital setting.

FAIT is such a RCT comparing arthroscopy versus physiotherapy rehabilitation for the management of femoroacetabular impingement in young adults. The trial is ongoing and aims to recruit a maximum of 214 participants by July 2016. Currently, 130 participants (at least 60% of maximum target) have been recruited. There are five participating centres showing variability in the recruitment rates. Feasibility work conducted prior to the trial concluded that 90% of eligible patients are willing to participate. However, the proportion of eligible patients opting-in is lower than that suggested by the feasibility study. The main factor identified as affecting recruitment and conduct of the trial is patient preference for surgery. Additionally, factors such as differences in waiting times, surgeon equipoise and fluctuating symptoms of the condition present significant constraints on planning the design and analysis for the trial. Alternate post-intervention design, treatment crossover, effective communication of treatment equipoise and careful site selection were considered to address these challenges. We highlight some unique challenges which influence conducting trials that specifically compare surgical intervention with rehabilitation. We aim to describe our experience and the decision making process involved in the planning and conduct of a pragmatic design for such a comparison in the context of the FAIT trial.

